# Exposure to acute normobaric hypoxia results in adaptions of both the macro- and microcirculatory system

**DOI:** 10.1038/s41598-020-77724-5

**Published:** 2020-12-01

**Authors:** Moritz Mirna, Nana-Yaw Bimpong-Buta, Fabian Hoffmann, Thaer Abusamrah, Thorben Knost, Oliver Sander, Yayu Monica Hew, Michael Lichtenauer, Johanna M. Muessig, Raphael Romano Bruno, Malte Kelm, Jochen Zange, Jilada Wilhelm, Ulrich Limper, Jens Jordan, Jens Tank, Christian Jung

**Affiliations:** 1 Division of Cardiology, Department of Internal Medicine II, Paracelsus Medical University of Salzburg, Muellner Hauptstrasse 48, 5020 Salzburg, Austria; 2 Department of Cardiology, Pulmonology and Vascular Medicine, Medical Faculty, Heinrich-Heine-University, Duesseldorf, Germany; 3 German Aerospace Center (DLR), Institute of Aerospace Medicine, Cologne, Germany; 4 Department of Cardiology, University Hospital Cologne, Cologne, Germany; 5 Department of Rheumatology, Hiller Research Institute for Rheumatology, Medical Faculty, Heinrich-Heine-University, Duesseldorf, Germany; 6 Department of Aeronautics and Astronautics, Stanford University, Stanford, CA 94305 USA; 7 Department of Anesthesiology and Intensive Care Medicine, Merheim Medical Center, Hospitals of Cologne, University of Witten/Herdecke, Cologne, Germany; 8 Chair of Aerospace Medicine, Medical Faculty, University of Cologne, Cologne, Germany

**Keywords:** Cardiology, Medical research, Pathogenesis, Hypoxia

## Abstract

Although acute hypoxia is of utmost pathophysiologic relevance in health and disease, studies on its effects on both the macro- and microcirculation are scarce. Herein, we provide a comprehensive analysis of the effects of acute normobaric hypoxia on human macro- and microcirculation. 20 healthy participants were enrolled in this study. Hypoxia was induced in a normobaric hypoxia chamber by decreasing the partial pressure of oxygen in inhaled air stepwisely (pO_2_; 21.25 kPa (0 k), 16.42 kPa (2 k), 12.63 kPa (4 k) and 9.64 kPa (6 k)). Macrocirculatory effects were assessed by cardiac output measurements, microcirculatory changes were investigated by sidestream dark-field imaging in the sublingual capillary bed and videocapillaroscopy at the nailfold. Exposure to hypoxia resulted in a decrease of systemic vascular resistance (*p* < 0.0001) and diastolic blood pressure (*p* = 0.014). Concomitantly, we observed an increase in heart rate (*p* < 0.0001) and an increase of cardiac output (*p* < 0.0001). In the sublingual microcirculation, exposure to hypoxia resulted in an increase of total vessel density, proportion of perfused vessels and perfused vessel density. Furthermore, we observed an increase in peripheral capillary density. Exposure to acute hypoxia results in vasodilatation of resistance arteries, as well as recruitment of microvessels of the central and peripheral microcirculation. The observed macro- and microcirculatory effects are most likely a result from compensatory mechanisms to ensure adequate tissue oxygenation.

## Introduction

The microcirculatory system comprises a network of small blood vessels with a pivotal role in maintaining adequate tissue perfusion, oxygenation and nutrient supply at the cellular level. Anatomically, the network consists of arterioles, venules and capillaries, with diameters well below 100 µm^1^. Current evidence suggests that the microcirculation plays a paramount role in the pathophysiology of multi-organ failure in critically ill patients, which is why the evaluation of microcirculatory disorders is gaining increasing recognition in intensive care medicine^2^. In fact, reduced cardiac output, changes in peripheral vascular resistance or alterations of the volume status or pH-value can lead to microcirculatory disorders, which result in tissue edema^3,4^, inadequate tissue perfusion and, subsequently, reduced cellular oxygen supply^2,5^. The resulting tissue hypoxia markedly aggravates tissue damage and thus promotes end-organ dysfunction in critically ill patients with sepsis or shock^6^, which is why the restoration of tissue perfusion and oxygenation constitutes a paramount treatment goal in clinical practice^5^.

Tissue hypoxia resulting from an inadequate uptake of ambient oxygen or an increase in cellular oxygen demand is one of the key features of the critically ill patient^7^. Since hypoxaemia, defined as a decrease in arterial oxygen tension^8^, is also a predominant feature of the high-altitude environment, research on the pathophysiologic processes behind hypoxia was significantly facilitated with the advent of altitude simulation tests^9^. On the cellular level, hypoxic stress initiates a transcriptional response by hypoxia inducible factors (HIF; during intermittent hypoxia predominately HIF-1α^10–12^), which leads to a reduction of cellular energy consumption, a secretion of pro-angiogenic and survival factors^10^, and qualitative changes in mitochondrial function^13^, which in turn results in alterations of the cardiovascular, haematological and even urinary physiology^14,15^. Among the observed physiological alterations in response to hypoxia, the effects on the human macrocirculatory system have been subject to several extensive scientific investigations in the past. Hence, acute hypoxia is known to result in an initial increase in heart rate, blood pressure and cardiac output, whereas a decrease in stroke volume can be observed only after a few days of exposure^16–18^. In contrast, studies concerning the effects of hypoxia on the microcirculatory system are comparatively scarce. For example, previous studies reported an increase in sublingual microcirculatory blood flow and capillary density after ascent to high altitudes^19,20^, which suggests microvascular recruitment after exposure to hypobaric hypoxia^21^. However, recent studies also reported that the physiological adaptions to hypobaric hypoxia can differ substantially from those to normobaric hypoxia^22,23^, which is why the results of studies conducted in high altitude can not be fully applied to the normobaric environment.

**Figure 1:**
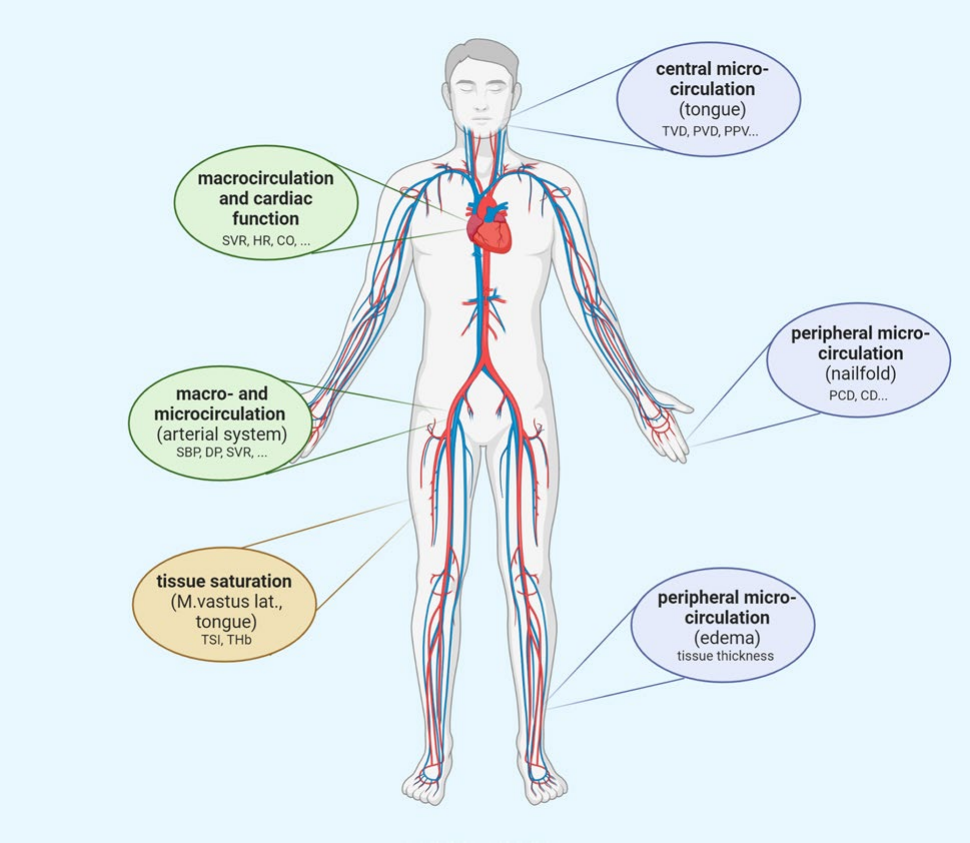
Schematic representation of the conducted measurements during the two hypoxia tests. *TVD* total vessel density, *PPV* proportion of perfused vessels, *PVD* perfused vessel density, *PCD* peripheral capillary recruitment, *CD* peripheral capillary diameter, *SV* stroke volume, *HR* heart rate, *CO* cardiac output, *SBP* systolic blood pressure, *DBP* diastolic blood pressure, *SVR* systemic vascular resistance, *TSI* tissue saturation index, *THb* total hemoglobin concentration.

Since the microcirculation constitutes one of the central components where hypoxia mediates its unfavourable effects in critically ill patients, a thorough investigation of the effects of normobaric hypoxia on the microcirculatory system, with regards to its interplay with larger vessels, is of interest. To further elucidate this matter, we conducted an altitude simulation test and investigated both the macro- and microcirculatory effects of acute normobaric hypoxia (Fig. 1 provides an overview of the conducted measurements).

## Results

In total, we enrolled 20 healthy subjects in this study, who had no significant experience in climbing or competitive sports. Of the subjects enrolled, the majority was male (n = 11, 55%), the median age was 29 years (IQR 25–31) and the median body mass index (BMI) was 23 kg/m^2^ (IQR 21–25.3). At baseline, the median systolic blood pressure (SBP) was 115 mmHg (IQR 106–128), the median diastolic blood pressure (DBP) was 70 mmHg (IQR 64.5–75), the median heart rate (HR) was 67 beats per minute (bpm; IQR 61.5–70), the median peripheral oxygen staturation (SpO_2_) was 97% (IQR 96–98) and the median respiratory rate was 16 min^−1^ (IQR 14–17, see Table 1). Eighteen subjects completed the entire hypoxia protocol.

**Table 1:** Baseline characteristics of the subjects enrolled.

	%	n (total = 20)
Sex (% male)	55	11

	Median	IQR
Age (years)	29	25–31
BMI (kg/m^2^)	23	21–25.3
Height (cm)	176.5	169.5–181.3
Weight (kg)	71.5	63.8–78.5
Systolic blood pressure (mmHg)	115	106–128
Diastolic blood pressure (mmHg)	70	64.5–75
Heart rate (bpm)	67	61.5–70
Respiratory rate (min^−1^)	16	14–17
Peripheral oxygen saturation (%)	97	96–98

The atmospheric data of the two hypoxia runs are displayed in Supplementary Figure 1. Briefly, the partial pressure of oxygen (pO_2_) in ambient air decreased significantly throughout the two hypoxia runs, whereas humidity, temperature, overall pressure and the partial pressure of carbon dioxide (pCO_2_) remained relatively stable.

Regarding symptoms of acute mountain sickness (AMS), the median Lake Louise Score (LLS) at baseline was 0.2 points (IQR 0.0–0.6), with a gradual increase to the median LLS of 3.8 points (IQR 1.7–4.4) at 6 k.

**Figure 2:**
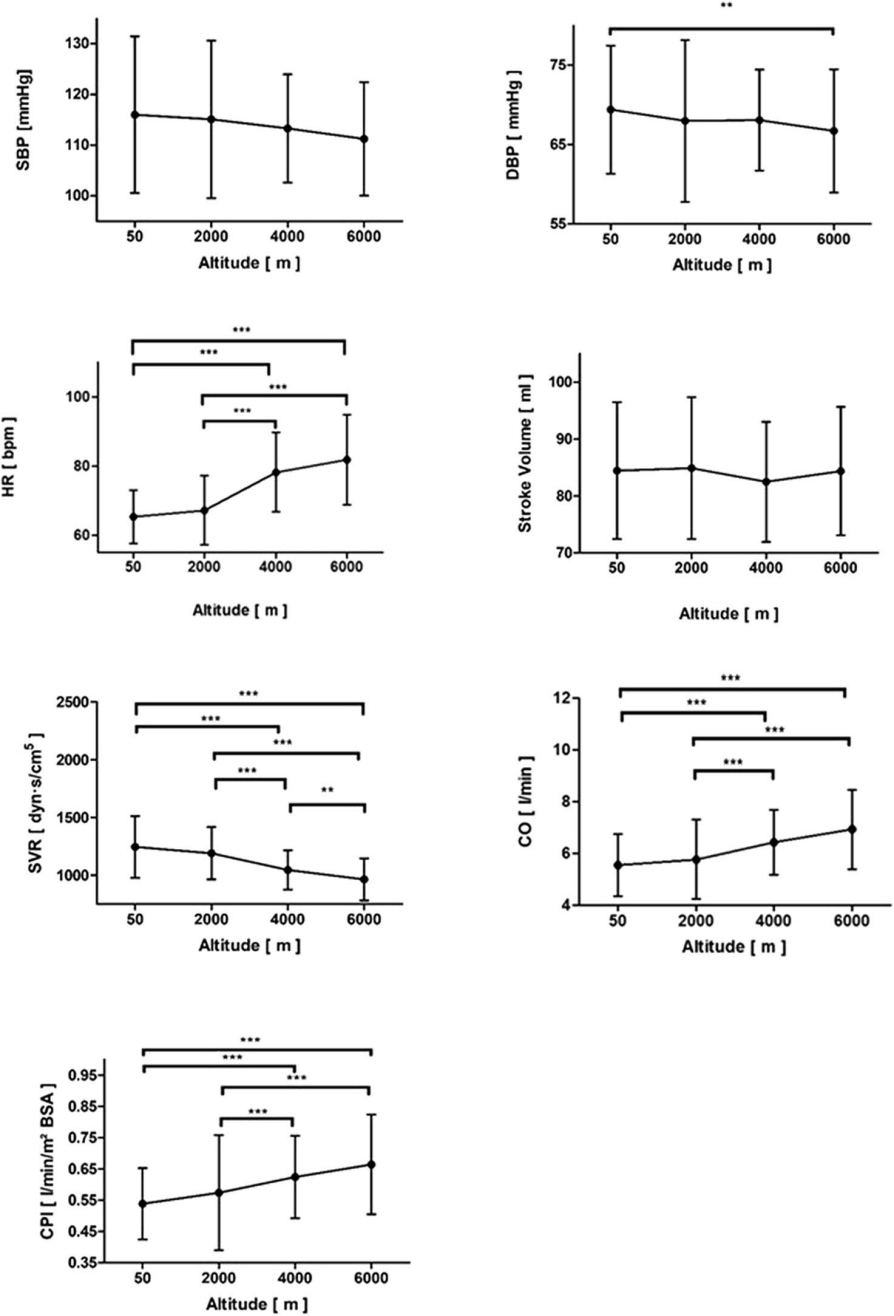
Systolic blood pressure (SBP), diastolic blood pressure (DBP), heart rate (HR), stroke volume (SV), systemic vascular resistance (SVR), cardiac output (CO) and cardiac performance index (CPI) throughout the altitude simulation test. *p < 0.05, **p < 0.01 and ***p < 0.001.

Exposure to hypoxia resulted in a significant decrease of DBP, but it did not result in a change of SBP. Whereas HR increased significantly throughout the two tests, stroke volume remained unchanged. Systemic vascular resistance decreased, whereas cardiac output (CO) and cardiac performance index (CPI) increased significantly (see Fig. 2, Table 2 and Suppl. Figure 2). Furthermore, exposure to hypoxia resulted in a significant decrease of SpO_2_ and a signficant increase in respiratory rate, as expected (see Table 2). Notably, there was no change in oxygen delivery (DO_2_), although a trend towards an initial decrease, followed by an increase to 6 k, was observed (see Table 2).

**Table 2:** Investigated variables throughout the altitude simulation tests.

	50 m (0 k)		2000 m (2 k)		4000 m (4 k)		6000 m (6 k)		*p* value
	Median	IQR	Median	IQR	Median	IQR	Median	IQR	
**Hemorheological variables**									
Red blood cell count (per pL)	4.9	4.3–5.5	4.9	4.2–5.4	4.9	4.3–5.4	4.9	4.3–5.3	0.984
Hemoglobin (g/dl)	14.8	12.9–15.7	14.4	13.0–15.7	14.5	12.8–15.5	14.6	12.7–15.5	0.944
Arterial oxygen content (ml/dl)	20.7	18.2–22.2	19.2	17.1–21.7	17.3	15.3–19.0	15.7	13.6–17.4	< 0.0001
Oxygen delivery (ml/min)	1105	971.8–1189	1069	950.4–1209	1040	916.4–1139	1128	977.2–1255	0.455
**Macrocirculation**									
Peripheral oxygen saturation (%)	97	97–97.5	95	93–97	84	80–87	80	67–83.5	< 0.0001
Respiratory rate (breaths per minute)	16	14–16	18	16–18	18	16–22	24	21–28	< 0.0001
Systolic blood pressure (mmHg)	115	105–130	114	103–122	116	103–119	110	104–122	0.251
Diastolic blood pressure (mmHg)	70	63–76	68	58–75	66	64–70	65	63–74	0.014
Systemic vascular resistance (dynes*s/cm^5^)	1206	1072–1421	1199	982–1352	1067	951–1152	940	814–1125	< 0.0001
Heart rate (bpm)	65	60–70	66	61–72	76	69–86	80	73–92	< 0.0001
Stroke volume (ml)	83	77–91	84	78–93	82	76–89	87	78–93	0.281
Cardiac output (l/min)	5.35	4.60–6.08	5.56	4.80–6.40	6.00	5.60–7.00	7.20	5.60–8.10	< 0.0001
Cardiac performance index (l/min/m^2^ BSA)	0.51	0.48–0.59	0.53	0.48–0.63	0.60	0.54–0.69	0.67	0.53–0.75	< 0.0001
**Sublingual microcirulation**									
Perfused number of crossings	32	27–37	33	27–40	35	29–39	36	30–42	< 0.0001
Perfused vessel density (mm/mm^2^)	6.8	5.7–7.8	7.0	5.7–8.5	7.4	6.2–8.3	7.7	6.4–8.9	< 0.0001
Proportion of perfused vessels (%)	94	89–97	94	89–98	95	90–98	95	91–100	0.017
Number of crossings	34	30–39	36	30–42	37	31–42	39	32–44	< 0.0001
Total vessel density (mm/mm^2^)	7.2	6.4–8.3	7.7	6.4–8.9	7.9	6.6–8.9	8.3	6.9–9.4	< 0.0001

	Mean	SEM	Mean	SEM	Mean	SEM	Mean	SEM	*p* value
**Peripheral microcirculation**									
Vessel density (n/mm^2^)	6.69	1.51	7.26	1.72	7.07	1.94	7.29	1.74	< 0.01
Peripheral capillary recruitment (%)	78.33	0.15	84.72	0.15	83.01	0.17	83.45	0.14	< 0.01
Peripheral capillary diameter, apex (µm)	16.58	7.77	17.32	8.09	16.99	8.26	17.14	8.12	0.01

	Baseline						Post hypoxia		*p* value
	Median	IQR					Median	IQR	
**Peripheral edema (tissue thickness)**									
Leg (mm)	5.14	4.4–7.0					5.54	4.7–6.4	0.936
Forehead (mm)	4.85	4.4–5.8					4.98	4.4–5.6	0.184

	Mean	SEM	Mean	SEM	Mean	SEM	Mean	SEM	*p* value
**Tissue oxygen saturation**									
Tissue saturation index tongue (%)	63.57	1.27	61.51	0.90	58.31	1.10	59.66	0.98	0.044
Tissue saturation index M.vastus lat. (%)	69.59	0.81	69.58	0.62	68.23	0.97	66.37	1.10	0.145
Total hemoglobin tongue (µmol/l)	129.5	4.14	136.3	5.56	123.1	6.91	123.8	7.24	0.356
Total hemoglobin M.vastus lat. (µmol/l)	48.27	3.94	46.83	3.56	49.57	4.48	48.43	4.20	0.997

**Figure 3:**
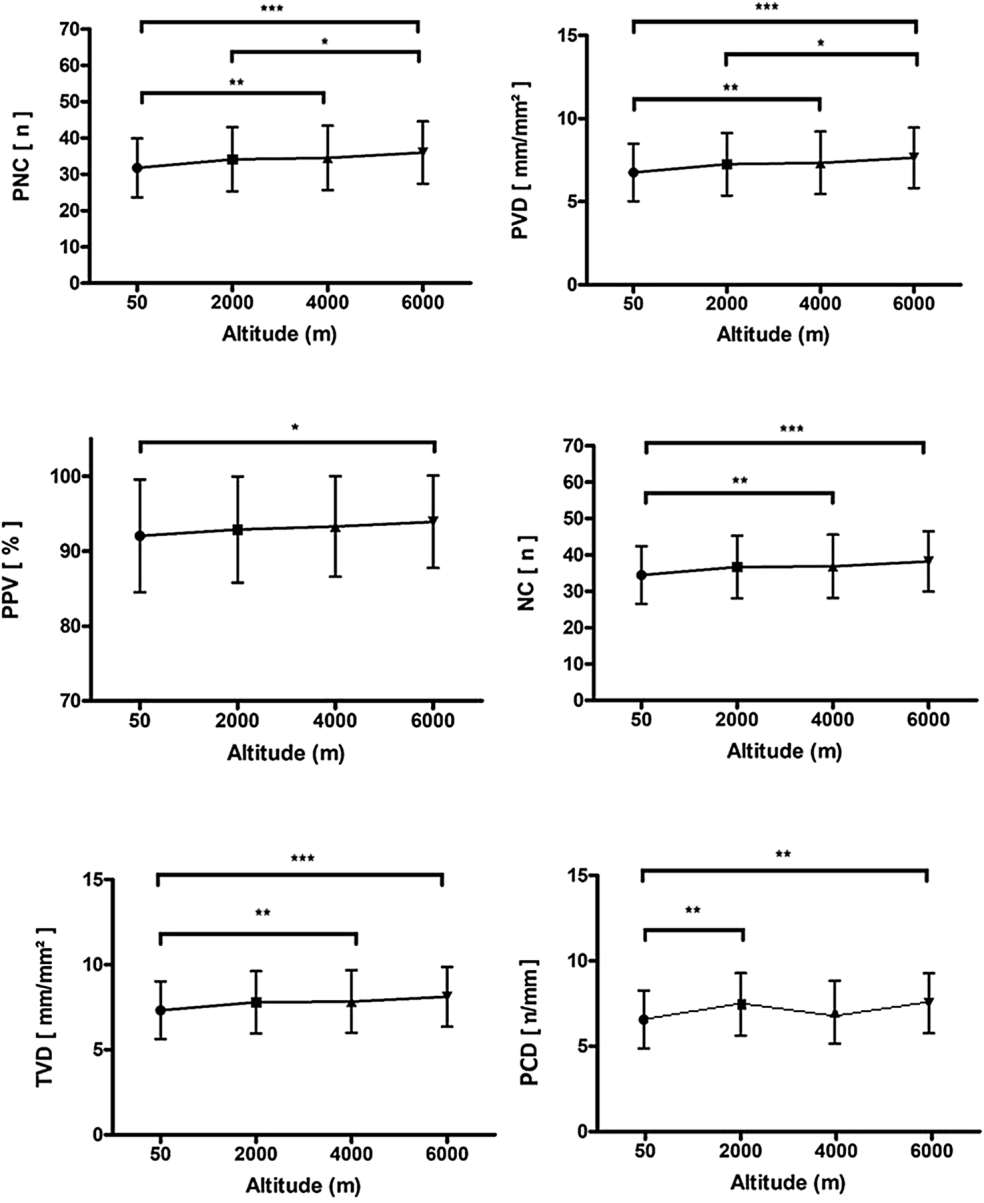
Perfused number of crossings (PNC), perfused vessel density (PVD), proportion of perfused vessels (PPV), number of crossings (NC), total vessel density (TVD), and peripheral capillary recruitment (PCD) throughout the altitude simulation test. *p < 0.05, **p < 0.01 and ***p < 0.001.

In the sublingual microcirculation, acute hypoxia resulted in an increase in the number of crossings (NC), total vessel density (TVD), perfused number of crossings (PNC), proportion of perfused vessels (PPV) and perfused vessel density (PVD; see Fig. 3, Table 2 and Suppl. Figure 2).

In the capillary bed of the nailfold, we observed a significant increase in peripheral capillary recruitment (PCD) at 2 k and 4 k when compared to the baseline values (0 k mean: 78.33% vs. 2 k: 84.72% and 4 k: 83.01%, *p* < 0.01, see Fig. 3, Table 2 and Supplementary Figure 3). Correspondingly, the mean peripheral capillary diameter (CD) of arterial limb (11.6 μm at 0 k), apex (16.6 μm at 0 k) and venous limb (15.7 μm at 0 k) showed a significant increase of 4% at 2 k (all *p* < 0.05) and 2% at 4 k (apex significant at *p* = 0.049). As estimated by the law of Hagen-Poiseuille^24^, which states that the flow rate is proportional to the radius of the vessel to the fourth power, the average flow increases corresponded to 17% (2 k) and 8% (4 k) and additionally 5% due to the increased capillary recruitment. Notably, the initial increase in PCD and CD was followed by a decrease in both variables at 6 k (see Table 2). A graphical overview of the findings concerning the macro- and microcirulatory system is provided in Supplementary Figure 2.

Changes in peripheral microcirculation did not result in clinical signs of increased microvascular permeability, since we observed no significant peripheral edema after hypoxia, as assessed by ultrasonographic tissue thickness of the lower leg or the forehead (see Table 2).

**Figure 4:**
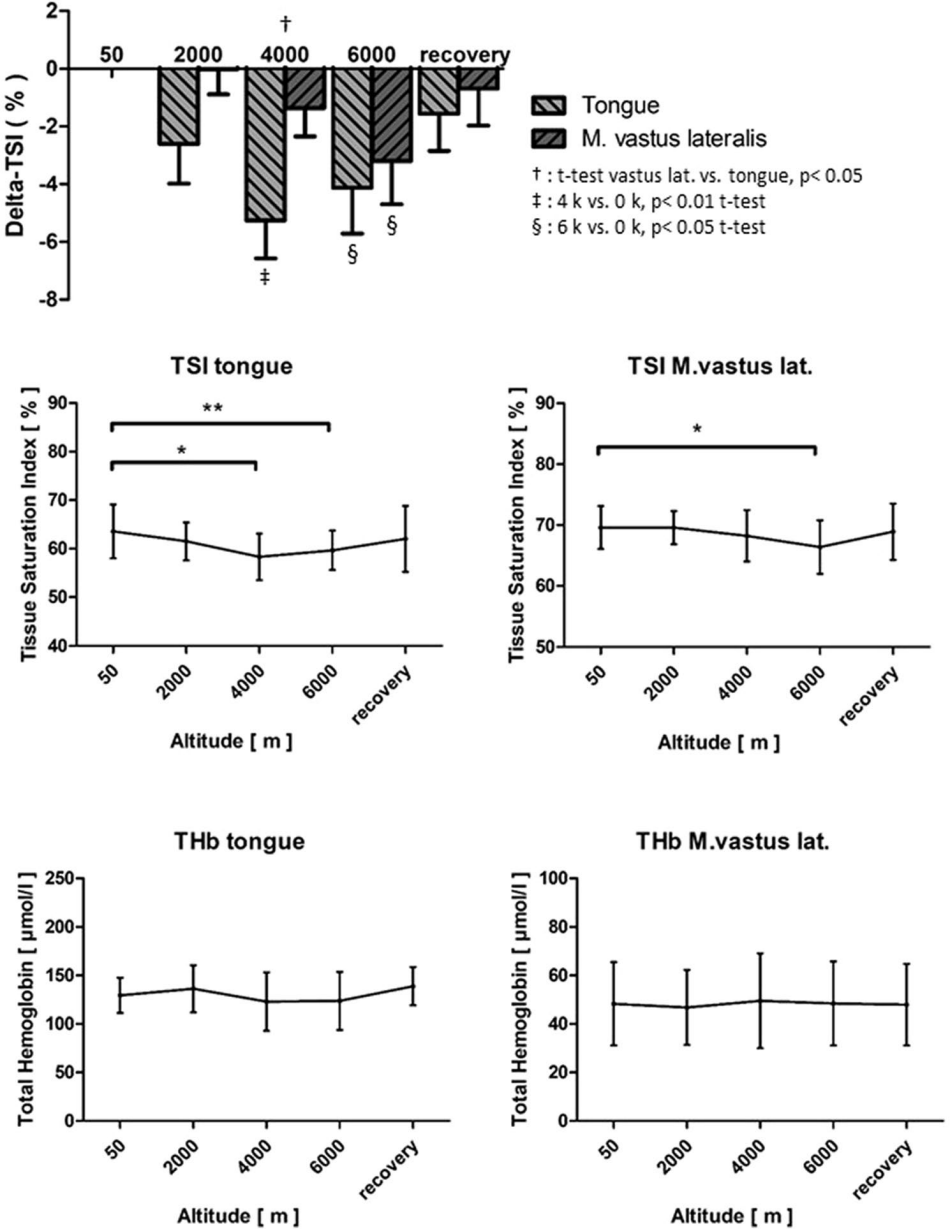
Tissue saturation index (TSI), Delta-TSI and total hemoglobin concentration (THb) of the tongue and the vastus lateralis muscle throughout the hypoxia test. † denotes the t-test of vastus lat. versus tongue at 4 k, where the *p* value was < 0.05; ‡ denotes the t-tests of the tongue and vastus lat. at 4 k versus 0 k, where the *p* value was < 0.01; § denotes the t-test of the tongue at 6 k versus 0 k, where the *p* value was < 0.05. *p < 0.05, **p < 0.01 and ***p < 0.001.

Concerning tissue oxygenation, we found only moderately diverging baseline values of the tissue saturation index (TSI) in both muscles at rest (see Table 2), but total hemoglobin concentration (THb) was almost three times higher in the tongue than in the vastus lateralis muscle. Similar to the decrease in SpO_2_, hypoxia resulted in a statistically significant, yet comparatively low change of the TSI of the tongue at 4 k and 6 k (baseline: mean 63.6% to 4 k: mean 58.3%, *p* = 0.01, and 6 k: mean 59.7%, *p* = 0.009) and of the vastus lateralis muscle at 6 k (baseline: mean 69.6% to 6 k: mean 66.4%, *p* = 0.013) when compared to the respective baseline values. In the tongue, however, the decrease in TSI in reponse to hypoxia reached statistical significance at more moderate levels of hypoxia and was in total more pronounced than in the vastus lateralis muscle. The THb was not significantly influenced by exposure to hypoxia (see Fig. 4 and Table 2). Also, the systemic red blood cell counts (RBC) and hemoglobin concentration remained unchangend (see Table 2).

In the two hypoxia runs conducted, a total of 2 subjects had to exit the test prematurely because of severe symptoms of AMS. These two participants did not show any difference in the investigated macro- and microcirculatory variables when compared to participants who did not exit the tests prematurely.

Participants who showed objective hypoxia, as portrayed by an SpO_2_ < 75% at 6 k (SpO_2_ < 75%: n = 8) had a significantly higher LLS at 2 k (median 1.7 vs. 0.5, *p* = 0.015), but a significantly lower respiratory rate at 6 k (median 21.3 breathspm vs. 27.7 breathspm *p* = 0.026). Moreover, the increase in HR from baseline to 6 k was significantly higher in these patients (Delta HR at 6 k: 23.8 vs. 10.1, *p* = 0.036).

Supplementary Table 1 depicts an overview of the differences between female and male participants, while the rest of the investigated variables were not different between the two genders. Compared to male participants, we observed a significantly lower SBP, DBP and THb of the vastus lateralis muscle in female subjects.

## Discussion

In the last decades, several studies have investigated the effects of hypoxia on the human macrocirculation. According to current evidence, chronic hypoxia leads to a significant increase of systolic and diastolic blood pressure by an overstimulation of the adrenergic and renin-angiotensin system^25,26^, as well as a downregulation of endothelial NO synthase (eNOS)^27^. Hence, long-term hypoxia is regarded a key precursor in the pathogenesis of arterial hypertension in patients with obstructive sleep apnea (OSA)^28^. In contrast, acute hypoxia is known to result in local or systemic vasodilatation via nitric oxide (NO), which is a direct result of enhanced secretion of adenosine, adenosine triphosphate (ATP), prostaglandins (PGs) and adrenaline^29^, and constitutes a compensatory mechanism to ensure adequate tissue perfusion^30,31^.

In our study, exposure to normobaric hypoxia resulted in profound systemic hypoxaemia, as portrayed by a significant decrease in SpO_2_. In contrast, decreases in TSI of the tongue and of the vastus lateralis muscle were comparatively low and probably clinically irrelevant. However, the response of TSI to hypoxia was more sensitive and pronounced in the tongue than in the leg muscle (Delta-TSI tongue min. − 5.3% at 4 k, Delta-TSI vastus lateralis muscle min. − 3.2% at 6 k, see Fig. 4), which indicates that hypoxia was predominantly compensated by an increased perfusion in both muscles at rest, with a more effective compensation in the vastus lateralis muscle, which is also adapted to high increases in energy turn-over during work.

Similar to previous studies^30,31^, we found that exposure to acute hypoxia resulted in a decrease of DBP. This finding was most likely a result from vasodilatation of the arterioles, as portrayed by a significant decrease in SVR. Concomitantly, we observed a compensatory increase in HR which, since there was no significant change in stroke volume, resulted in an increased CO (CO = HR × SV) and CPI.

The observed macrocirculatory effects of systemic hypoxaemia were accompanied by microcircultatory changes, which indicate an increase in organ perfusion. Hence, we found that exposure to normobaric hypoxia leads to a significant increase in TVD, PPV and PVD of the sublingual microcirculation, which can be interpreted as a result of capillary recruitment. Capillary recruitment is known as an opening of previously closed capillaries by dilatation of the precapillary sphincters in response to unmet metabolic demands^32^. In fact, only 20–30% of the capillaries are actively participating in tissue perfusion under resting conditions^33^. Therefore, capillary recruitment constitutes an important compensatory mechanism to ensure adequate tissue perfusion and oxygenation in the capillary beds of several muscles^34^ and the lungs^35^. Notably, our findings are in line with the results of a previous study by Hilty et al.^21^, who found that exposure to high altitudes was associated with capillary recruitment of sublingual capillaries and thus an increase in microcirculatory oxygen extraction capacity.

Similar to the microcirulation of the sublingual capillary bed, we observed a significant increase in PCD and CD in the microcirculation of the nailfold. This indicates capillary recruitment in the peripheral microcirculation by exposure to acute hypoxia. Notably, Paparde et al. recently reported that acute hypoxia does not influence capillary recruitment in human nailfold capillaries, but rather leads to capillary vasodilatation^36^. Since capillary recruitment is a rapid adaptation, the short duration of hypoxia in this study is no plausible explanation for the discrepancy of the study’s results to our current findings. In our study cohort, however, we were able to identify identical areas of the nailfold where we could demonstrate capillary recruitment directly (e.g. capillaries visible at 2 k that were not visible at 0 k, see Supplementary Figure 3) and could calculate recruitment rates fitting to the expected increase in circulation. Furthermore, in contrast to hypoxia, hyperoxia was recently found to reduce capillary recruitment^37^. Considering our current data and this previous finding, it seems plausible and credible that dysregulations of the pO_2_ in blood affect the microcirculation of the nailfold.

In the last decades, clinicians have increasingly recognized the role of the microcirculatory system in the pathophysiology of different diseases. In fact, current evidence suggests, that microcirculatory disorders are a key component of the processes involved in the pathogenesis of multi-organ failure in critically ill patients^2^. Hence, microcirculatory dysfunctions can be found in a large proportion of patients admitted to intensive care units. For example, a reduction in PPV and PVD can be observed in patients with sepsis, and can be interpreted as a result of the impairment of the functional perfusion of the microcirculation^6,38,39^. Microcirculatory disorders themselves can result in tissue hypoxia, which markedly aggravates end-organ dysfunction in critically ill patients^6^. In fact, microcirculatory changes could even be associated with adverse outcomes in several previous studies^40^, which is why the restoration of adequate tissue perfusion and oxygenation constitutes a paramount treatment goal in clinical practice.

The observed macro- and microcirculatory effects of acute hypoxia in healthy participants in our study are most likely a result from compensatory mechanisms which ensure adequate tissue perfusion in case of profound hypoxaemia. Hence, by recruiting microvessels of the central and peripheral microcirculation, the organism adapts to hypoxaemia to counteract the state of inadequate tissue oxygenation. In fact, tissue hypoxia resulting from inadequate uptake of ambient oxygen or an increase in cellular oxygen demand is one of the key features of the critically ill patient^7^. However, whether the observed compensatory mechanisms also occur in the hypoxaemic patient, and, if absent or reduced microcirculatory adaptions are associated with adverse outcomes, remains to be elucidated in clinical trials.

## Materials and methods

The study protocol of this exploratory study was reviewed and approved by the ethics committee of the Heinrich-Heine-University, Düsseldorf, Germany (5925R) and conducted according to the principles of the Declaration of Helsinki and Good Clinical Practice. Informed consent was obtained from all subjects before enrollment.

The study was conducted at the ‘DLR:envihab’ of the German Aerospace Center (https://www.dlr.de/envihab/), Cologne, Germany. In total, 20 healthy subjects without significant experience in climbing or competitive sports were enrolled by an announcement at the University of Düsseldorf. The anthropometric data and baseline characteristics of the study participants are depicted in Table 1.

### Induction of hypoxia, measurement of oxygen delivery and Lake Louise Score (LLS)

Baseline measurements of 20 healthy participants were acquired under normoxic conditions and compared to measurements under normobaric hypoxic conditions. Hypoxia tests were performed in a normobaric hypoxia chamber, which comprises a laboratory space of about 120 m^2^ including examination rooms, sanitary facilities and a big common room where subjects could move freely and where they waited for individual examinations. Hypoxia was achieved by nitrogen dilution through the air conditioning system in the atmoshperic self-sufficient hypoxia chamber. Nitrogen was supplied by an external tank. Hence, four different altitudes above sea level were simulated (50 m (0 k), 2000 m (2 k), 4000 m (4 k) and 6000 m (6 k) above sea level). Throughout the test, the partial pressure of nitrogen in ambient air was gradually increased, which led to a reduction of the partial pressure of oxygen (pO_2_; 21.25 kPa (0 k), 16.42 kPa (2 k), 12.63 kPa (4 k) and 9.64 kPa (6 k), FiO2: median 0.21 (0 k), 0.16 (2 k), 0.12 (4 k), 0.10 (6 k), see Supplementary Figure 1). The partial pressure of carbon dioxide remained relatively stable throughout the two hypoxia runs (pCO_2_ median 0.046 kPa (0 k), 0.060 kPa (2 k), 0.72 kPa (4 k) and 0.059 kPa (6 k)). The subjects were exposed to an ‘altitude’ for two hours before proceeding to the next level. Measurements were performed at each oxygen level to investigate the effects of hypoxia on the micro- and macrocirculation (Fig. 1 provides an overview).

Oxygen delivery (DO_2_) was calculated as previously published (DO_2_ = cardiac output (CO) × CaO_2_ × 10; where CaO_2_ was the arterial O_2_-content defined as: (1.34 × hemoglobin concentration × SpO_2_) + (0.003 × PaO_2_) and the amount of dissolved oxygen in blood was estimated by 0.003 × pO_2_)^41^. Hemoglobin concentration and red blood cell count (RBC) were measured at each of the four different altitudes using an ABL800 FLEX blood gas analyzer (Radiometer Medical, Copenhagen, Denmark).

Symptoms of hypoxia and severity of acute mountain sickness (AMS) were assessed by the Lake Louise Score (LLS), as previously published. Mild AMS was defined as LLS 3–5 points, moderate AMS as 6–9 points, and severe AMS as 10–12 points^42^.

### Macrocirculation and cardiac function

Systolic and diastolic blood pressure (SBP and DBP) were assessed using a ProBP 3400 (Welch Allyn, Skaneateles Falls, New York, USA) blood pressure monitor, peripheral oxygen saturation was assessed using a PULOX PO-300 (Novidion, Cologne, Germany) pulse oximeter. Systemic vascular resistance (SVR), heart rate (HR), cardiac output (CO), cardiac performance index (CPI) and stroke volume (SV) were measured using transthoracic impedance cardiography (ICON, OSYPKA Medical, Berlin, Germany) and non-invasive Electrical Cardiometry (EC, OSYPKA Medical, Berlin, Germany).

### Central and peripheral microcirculation

A sidestream darkfield microscope (MicroScan device, MicroVision Medical, Amsterdam, The Netherlands) was used to assess the sublingual microcirculation as described previously^43^. Only sufficiently trained researchers performed measurements. In brief, on the tip of this device, a highly sensitive camera digitally recorded the sublingual capillary network. Different regions under the tongue were used for all videos and at least four videos were taken per area. In the next step, a tablet computer was used (Microsoft Surface Pro 4, (Redmond, Washington, USA)) for video analysis. After recording videos with sufficient quality, a validated automatic algorithm-software (AVA, Version 4.3 C, MicroVision Medical, Amsterdam, The Netherlands) was used to perform the offline analysis. Agreeing to the second consensus on the assessment of sublingual microcirculation in critically ill patients (European Society of Intensive Care Medicine), the following variables were assessed^44^:

Microvascular values can offer information about both convexity and diffusion. The proportion of perfused vessels (PPV = 100 * (Total number of perfused vessels / total number of vessels) gives information both about convexity and perfusion. Diffusion can be assessed by the total vessel density (TVD) and the number of crossings (NC). Density was evaluated by the total vessel density and the number of crossings. With both information, the perfused vessel density (PVD = total length of perfused vessels divided by the analyzed area) and perfused number of crossings (PNC = number of vessel crossings with continuous flow) can be calculated. Vessels with diameters of less than 20 mm correspond mostly to capillaries and are primarily responsible for the microcirculation. These small vessel values are signed with the prefix “s” (e.g. sPPV = PPV of small vessels). The values for all vessels can be considered as a quality check to exclude for example pressure artifacts. Before AVA 4.3C analysis, all videos were evaluated according to the microcirculation image quality score (MIQS), that were originally introduced by Massey et al.^45^. In brief, MIQS rates the acquired videos into three categories: “good”, “acceptable”, and “non-acceptable”. Overall, six different criteria are evaluated: illumination, focus, content, stability, pressure, and the duration was not rated. A video without significant impairment in all criteria gets zero points. Mild impairment results in 1 point for each impaired criterion. Severe impairment in one criterion is defined to be rated with 10 points, which results in the category “non-acceptable”.

Peripheral microcirculation at the nailfold was assessed at two areas of fingers III-V of both hands, images of the nailfold of 1 mm were obtained using videocapillaroscopy (Di-Li 2100, Distelkamp-Electronic, Kaiserslautern, Germany). The acquired images were assigned to each other and identical capillaries were compared in density (PCD), visibility (visible 1, scanty 0.5, not visible 0, not shown x), shape (hairpin 1, tortuous 2, abnormal 3), distance (to the right capillary 50 μm before apex in μm), diameter (CD) of the arterial limb (50 μm before apex in μm), apical (in the middle in μm), venous limb (50 μm behind apex in μm) and tail (diameter between arterial and venous limb 50 µm from apex in μm).

Assessment of the microcirculation was conducted according to the current recommendations of the European Society of Intensive Care Medicine^44^.

### Peripheral microcirculation and edema

To investigate whether microcirculatory disorders resulting from generalized hypoxia cause peripheral edema, we performed ultrasonographic measurements of the tissue thickness of the leg and the forehead in supine position. Measurements were conducted in the evening before the hypoxia tests and immediately after the subject left the hypoxic atmosphere. Tissue thickness was measured repeatedly by ultrasound (10–18 MHz linear-probe) at both the anterior tibia and the midline forehead locations (MyLab25, Esaote, Genova, Italy). In more detail, a self-made mechanical fixture device was used to avoid measurement bias due to hand-held measurements and different surface pressure of the ultrasound probe on tissue. In addition an automatic, Matlab (Matlab 2017b, The MathWorks Inc., Natick, USA) based tissue identification software was used to reliably estimate tissue thickness. The software enables automatic and robust tissue thickness estimation from the ultrasound images to minimize analysis biases and improves analysis efficiency during tissue thickness identification and evaluation. The average tissue thickness was calculated from the central section of the ultrasound image (only 50% of the image at the center) to reduce calculation bias caused by the imaging technique. Tissue thickness was defined as the distance between the surfaces of the skin and the tibia and frontal bone, respectively.

### Tissue oxygenation

We investigated the effects of hypoxia on oxygen saturation and blood content of the muscles by assessing the tissue saturation index (TSI = 100*(O_2_Hb)/(O_2_Hb + (HHb); O_2_Hb = oxygenated hemoglobin, HHb = desoxygenated hemoglobin) and the content of total hemoglobin (THb = O_2_Hb + HHb) in the vastus lateralis muscle and the tongue by near-infrared spectroscopy (NIRS) at each of the different altitude levels and after the end of the hypoxia tests. For the examination of vastus lateralis muscle we placed a PortaMon device (Artinis Medical Systems, Elst, The Netherlands) over the belly of the muscle. For measuring the tongue we used the PortaLite device (Artinis Medical Systems, Elst, The Netherlands) placing the sensor part on the tongue after shielding it from saliva fluid using a thin plastic foil.

### Statistical analysis

Statistical analysis was conducted using GraphPad Prism software (GraphPad Software, USA) and SPSS (Version 24.0, SPSSS Inc., USA). Normally distributed data were expressed as mean and standard error of the mean (SEM), whereas not normally distributed data were shown as median and interquartile range (IQR). A Kolmogorov–Smirnov test was used to test the distribution of data for normality. Medians of the data on micro- and macrocirculation were analyzed using a Kruskal–Wallis test with Dunn’s post-hoc test, data on the tissue thickness were analyzed using a Wilcoxon signed-rank test, and data on the TSI and THb of the muscles were interpreted by applying a Student’s t-test and a Linear Mixed Effects (LME)-analysis. A *p* value < 0.05 was considered statistically significant.

### Ethics approval

The study was reviewed and approved by the ethics committee of the Heinrich-Heine-University, Düsseldorf, Germany (5925R) and conducted according to the principles of the Declaration of Helsinki and Good Clinical Practice. Informed consent was obtained from all subjects before enrollment.

## Conclusions

Exposure to acute normobaric hypoxia results in enhanced perfusion of the central and peripheral microcirculation, as well as an increase in cardiac output. The observed macro- and microcirculatory effects are most likely a result from compensatory mechanisms to ensure adequate tissue perfusion in case of profound hypoxaemia.

## Limitations

Since this study was an exploratory study, we did not conduct a statistical power calculation prior to enrollment. Hence, a type one error can not certainly be excluded. Another limitation is that SVR, CO, CPI and SV were investigated using transthoracic impedance cardiography, which is less accurate than invasive methods. Due to the technique of impedance cardiography, which is based on calculations derived from the basic laws of electricity, several limitations and possibilities for bias arise^46,47^, which have to be taken into consideration when interpreting the findings of our study. However, invasive methods, such as thermodilution, are labor- and time-intensive and unsuitable for the narrow setting of a hypoxia chamber. Hence, we chose transthoracic impedance cardiography over invasive methods in our study.

## Perspectives

Herein, we provide a comprehensive analysis of the effects of acute normobaric hypoxia on the macro- and microcirculation in healthy subjects. Our findings can contribute to the understanding of health and disease, since hypoxia and the following systemic reactions are of utmost relevance in the pathophysiology of various diseases.

## Supplementary information

Supplementary Legends.

Supplementary Information.

## Data Availability

The datasets generated during and analyzed during the current study are available from the corresponding author on reasonable request.

## Abbreviations

AMS: Acute mountain sickness
CaO_2_: Arterial oxygen content
CD: Peripheral capillary diameter
CO: Cardiac output
CPI: Cardiac performance index
DBP: Diastolic blood pressure
DO_2_: Oxygen delivery
FiO_2_: Fraction of inspired oxygen
kPa: Kilopascal
HR: Heart rate
LLS: Lake Louise Score
NC: Number of crossings
NIRS: Near-infrared spectroscopy
PCD: Peripheral capillary recruitment
pCO_2_: Partial pressure of carbon dioxide
PNC: Perfused number of crossings
pO_2_: Partial pressure of oxygen
PPV: Proportion of perfused vessels
PVD: Perfused vessel density
SBP: Systolic blood pressure
SV: Stroke volume
SVR: Systemic vascular resistance
THb: Total hemoglobin concentration
TSI: Tissue saturation index
TVD: Total vessel density
