# Supplementary material for: Exposure to acute normobaric hypoxia results in adaptions of both the macro- and microcirculatory system
Source: Sci Rep. 2020 Dec 1;10:20938. doi: 10.1038/s41598-020-77724-5 (PMC7708486; doi:10.1038/s41598-020-77724-5)
Supplement: Supplementary file 1 — Supplementary Legends. [file 41598_2020_77724_MOESM1_ESM.docx]

**Suppl. Table 1**: Statistically significant differences between male and female study participants in the investigated variables. *SBP= systolic blood pressure, DBP= diastolic blood pressure, HR= heart rate, CO= cardiac output, CPI= cardiac preformance index, SV= stroke volume, THb= total hemoglobin concentration*

**Suppl. Figure 1:** Atmospheric data of the two hypoxia tests conducted. *pO_2_= partial pressure of oxygen, FiO_2_= fraction of inspired oxygen, pCO2= partial pressure of carbon dioxide, kPa= kiloopascal*

**Suppl. Figure 2:** Schematic representation of the microcirculatory and macrocirculatory changes that were observed during the hypoxia tests.

**Suppl. Figure 3:** Capillaries of the same region representing 500 µm nailfold in width at 50 m (0 k) and 2000 m (2 k). The picture demonstrates the recruitment of two additional capillaries (B and E) at 2 k, which were not visible at 0 k.
