# Supplementary material for: Exposure to acute normobaric hypoxia results in adaptions of both the macro- and microcirculatory system
Source: Sci Rep. 2020 Dec 1;10:20938. doi: 10.1038/s41598-020-77724-5 (PMC7708486; doi:10.1038/s41598-020-77724-5)
Supplement: Supplementary file 2 — Supplementary Information. [file 41598_2020_77724_MOESM2_ESM.pdf]

Exposure to Acute Normobaric Hypoxia results in Adaptions of both the Macro- and Microcirculatory System

Supplementary Material

Moritz Mirna, MD[1*], Nana-Yaw Bimpong-Buta, MD[2*], Fabian Hoffmann, MD[3,4], Thaer Abusamrah[2], Thorben Knost[2], Oliver Sander, MD[5], Yayu Monica Hew,PhD[6], Michael Lichtenauer, MD, PhD[1], Johanna M Muessig, MD[2], Raphael Romano Bruno, MD[2], Malte Kelm, MD[2], Jochen Zange, PhD[3], Jilada Wilhelm[3], Ulrich Limper, MD[3,7], Jens Jordan, MD[3,8], Jens Tank, MD[3], Christian Jung, MD, PhD[2]

*contributed equally

[1] Department of Internal Medicine II, Division of Cardiology, Paracelsus Medical University of Salzburg, Austria
[2] Department of Cardiology, Pulmonology and Vascular Medicine, Medical Faculty, Heinrich-Heine-University Duesseldorf, Germany
[3] German Aerospace Center (DLR), Institute of Aerospace Medicine, Cologne, Germany
[4] Department of Cardiology, University Hospital Cologne, Germany
[5] Department of Rheumatology and Hiller Research Institute for Rheumatology, Medical Faculty, Heinrich-Heine-University Duesseldorf, Germany
[6] Stanford University, Department of Aeronautics and Astronautics, Stanford University, Stanford,CA 94305, ymhew@alumni.stanford.edu
[7] Department of Anesthesiology and Intensive Care Medicine, Merheim Medical Center, Hospitals of Cologne, University of Witten/Herdecke, Cologne, Germany
[8] Chair of Aerospace Medicine, Medical Faculty, University of Cologne, Cologne, Germany

Running head: Macro- and microcirculatory responses to hypoxia

Corresponding author:

Moritz Mirna, MD

Department of Internal Medicine II, Division of Cardiology
Paracelsus Medical University of Salzburg
Muellner Hauptstrasse 48
A-5020 Salzburg
Austria
Email: m.mirna@salk.at
Telephone: +43 (0) 57255 - 58340
ORCID iD: https://orcid.org/0000-0001-5679-4872

Conflict of interest disclosure: The authors declare that there is no conflict of interest regarding the publication of this paper.

Supplementary Figure 1

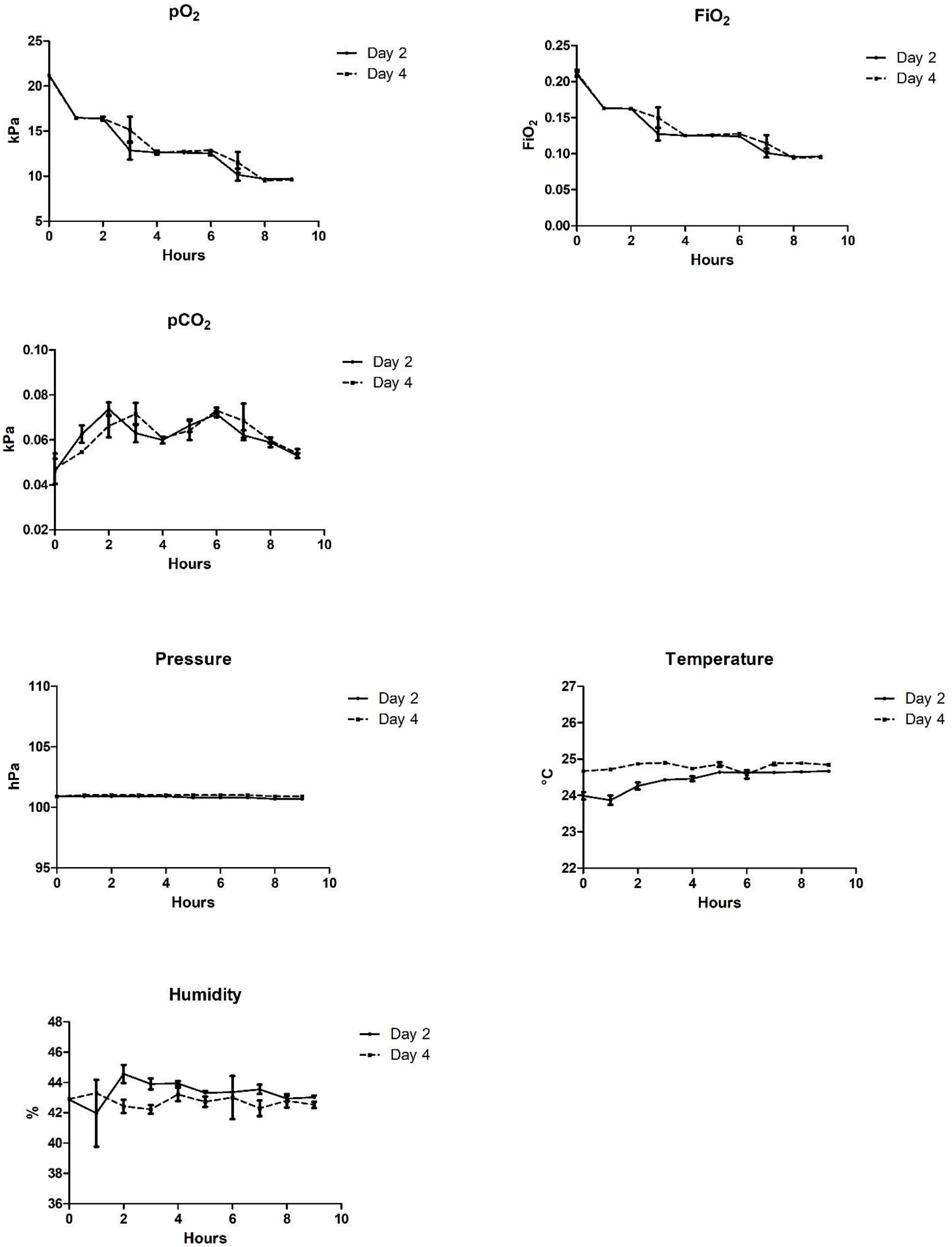

Supplementary Figure 2

Central Microcirculation	2000 m (2k)	4000 m (4k)	6000 m (6k)
Perfused number of crossings	↑	↑	↑
Perfused vessel density	↑	↑	↑
Proportion of perfused vessels	↔	↑	↑
Number of crossings	↑	↑	↑
Total vessel density	↑	↑	↑

Macrocirculation	2000 m (2k)	4000 m (4k)	6000 m (6k)
Peripheral oxygen saturation	↓	↓	↓
Respiratory rate	↑	↑	↑
Systolic blood pressure	↔	↔	↔
Diastolic blood pressure	↓	↓	↓
Systemic vascular resistance	↓	↓	↓
Heart rate	↑	↑	↑
Stroke volume	↔	↔	↔
Cardiac output	↑	↑	↑
Cardiac performance index	↑	↑	↑

Peripheral Microcirculation	2000 m (2k)	4000 m (4k)	6000 m (6k)
Peripheral capillary density	↑	↑	↔
Peripheral capillary diameter	↑	↑	↔
Tail diameter	↔	↔	↔

Supplementary Figure 3

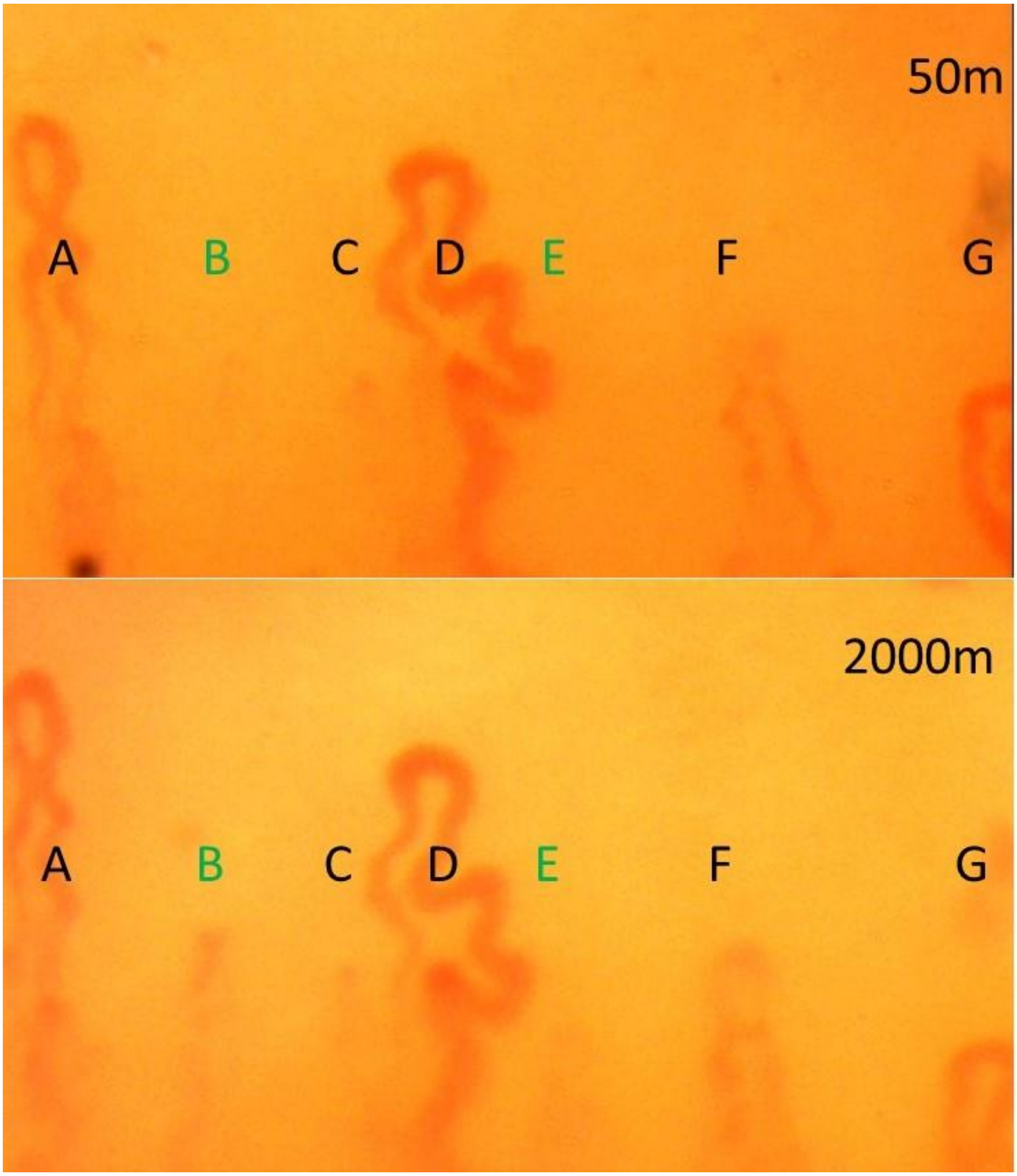

Supplementary Table 1

	Female (n= 9)		Male (n= 11)		
	median	IQR	median	IQR	p-value
SBP at baseline (mmHg)	105	97-108	128	115-136	< 0.0001
SBP at 2 k (mmHg)	103	98.5-108	122	118-138	< 0.0001
SBP at 4 k (mmHg)	104	98.5-109	119	116-126	< 0.0001
SBP at 6 k (mmHg)	104	97.8-109	117	109-123	0.021
DBP at baseline (mmHg)	63	59-71	75	70-78	0.002
DBP at 2 k (mmHg)	58	56-63	75	72-80	< 0.0001
DBP at 4 k (mmHg)	64	63-66	70	67-77	< 0.0001
DBP at 6 k (mmHg)	62	57-65	74	68-79	0.002
HR at 2 k (bpm)	63	57-66	87	80-96	< 0.0001
CO at 2 k (l/min)	4.95	4.36-5.99	6.18	5.27-7.82	0.017
CO at 4 k (l/min)	5.81	5.38-6.11	6.60	5.83-7.94	0.035
CPI at 2 k (l/min/m² BSA)	0.50	0.40-0.52	0.63	0.57-0.75	0.001
SV at baseline (ml)	77	68.6-82.2	89.1	79.6-92.8	0.043
THb tongue at 4 k (µmol/l)	119.1	93.0-127.6	135.0	128.2-145.5	0.006
THb vastus lateralis at baseline (µmol/l)	39.2	23.3-52.0	52.3	47.2-64.5	0.010
THb vastus lateralis at 2 k (µmol/l)	41.6	22.4-49.1	54.3	47.4-64.3	0.010
THb vastus lateralis at 4 k (µmol/l)	39.8	21.8-47.3	58.1	49.7-74.9	0.001
THb vastus lateralis at 6 k (µmol/l)	39.4	20.1-53.0	55.4	50.9-62.3	0.015
